# Optic nerve sheath diameter at high altitude: standardized measures in healthy volunteers

**DOI:** 10.1186/s13089-022-00295-1

**Published:** 2022-11-19

**Authors:** Edith Elianna Rodríguez Aparicio, Jorge Armando Carrizosa Gonzalez, David Rene Rodriguez Lima

**Affiliations:** 1 Critical and Intensive Care Medicine Department, Hospital Universitario Fundación Santa Fe de Bogotá, Bogotá, Colombia; 2 Faculty of Medicine and Health Sciences, Universidad del Rosario, Bogotá, Colombia; 3 Critical and Intensive Care Medicine, Hospital Universitario Mayor-Mederi, Bogotá, Colombia; 4 Grupo de Investigación Clínica, Escuela de Medicina y Ciencias de la Salud, Universidad del Rosario, Bogotá, Colombia

**Keywords:** Ultrasound, Optic nerve sheath diameter, Eyeball transverse diameter, Altitude, Intracranial pressure, Normal value, Normal population

## Abstract

**Background:**

Increases in the diameter of the optic nerve sheath (ONSD) on ultrasound are associated with high intracranial pressure (hICP). The normal value varies with altitude and the population studied. The objective of this study is to describe the normal values of the ONSD in a healthy adult population of the city of Bogotá, Colombia, at 2640 meters above sea level (masl).

**Patients and methods:**

A prospective observational study was conducted on a total of 247 healthy individuals recruited from May 2021 to May 2022 who were subjected to the color, low power, optic disk, safety, elevated frequency, dual (CLOSED) protocol for measuring the bilateral ONSD adjusted to the eyeball transverse diameter (ETD).

**Results:**

A total of 230 individuals were analyzed; the average ONSD of the right eye (RE) was 0.449 cm (range 0.288–0.7) and that of the left eye (LE) was 0.454 cm (range 0.285–0.698); the correlation between RE and LE was 0.93 (*p* < *0.005),* and the correlation of the ONSD/ETD ratios for the RE and LE was lower (*r*^2^ = 0.79, *p* < *0.005).* A total of 10.8% of the studied population had values greater than 0.55 cm.

**Conclusions:**

The median ONSD and ONSD/ETD ratio in the city of Bogotá are similar to those described in other populations; however, approximately 10.8% of the healthy population may present higher values, which would limit the use of ONSD on its own for clinical decision-making, only repeated measurements with significant changes in the ONSD and ONSD/ETD or asymmetries between the measurements of both eyes linked to clinical findings would allow the diagnosis of hICP.

## Background

The Monro–Kellie doctrine describes the behavior of intracranial pressure (ICP) based on the dynamics of 3 variables: blood volume (arterial and venous), cerebrospinal fluid (CSF) volume and cerebral parenchyma volume [1, 2, 3]. The total of the pressure of these three components within the cranial vault is interpreted as the ICP, which can be measured with invasive techniques that, although precise, can lead to tissue damage, while noninvasive methods are still in development [2, 4]

The diameter of the optic nerve sheath (ONSD) as measured on ultrasound assessment of the orbit has been shown to be an acceptable alternative for monitoring the ICP in patients in the intensive care unit [5, 6, 7, 8], since when interpreted as a continuum of the subarachnoid space through which the CSF can expand, it can enlarge in cases in which the other components of intracranial pressure are altered [9]. However, as most measurements performed by ultrasound have a certain degree of subjectivity, adjustment of the measurement by the ETD [10, 11] and the application of standardized protocols such as the color, low power, optic disk, safety, elevated frequency, dual (CLOSED) protocol [12] or the measurement from the internal and external layers of the optic nerve sheath that should be taken into account when making decisions in clinical practice [13].

Determining the presence of hICP from absolute values of the ONSD remains controversial due to the difficulty in establishing a cut-off for determining the increase in ICP. Values greater than 0.55 cm have been proposed in the literature for the diagnosis of hICP, with better correlations with elevated ICP obtained with values greater than 0.6 cm [14, 15]. The normal values vary according to the populations studied between 0.3 and 0.46 [16]. This great variability on the normal value of the ONSD and that adjusted by the ETD in healthy people may be related to hypobaric hypoxemia in cities at different altitudes above the sea level.

The objective of this study is to describe the pattern of normal values of the ONSD in a healthy adult population from the city of Bogotá, Colombia, at 2640 masl [17].

## Methodology

This was a prospective cohort study of an exploratory nature conducted from May 2021 to May 2022 that included the measurement of 247 healthy individuals who corresponded to the working staff, visitors, and students at the University Hospital of Fundación Santa Fe de Bogotá in the city of Bogotá, Colombia. Candidates for measurement were subjects older than 18 years who were at the institution as employees, students or visitors; who had lived in Bogotá for more than 6 months; who agreed to participate in the study and signed informed consent. People with a history of traumatic brain injury or ocular trauma, intracranial hypertension, ischemic or hemorrhagic cerebrovascular disease, neuroinfection, tumors at the level of the central nervous system or eyeball, previous optic nerve and ocular disease (glaucoma, uveitis or diseases that increase blood flow), epilepsy, recent facial trauma, pregnancy and hyperthyroidism were excluded from the measurements. The protocol for data collection and measurement was approved by the institutional ethics committee (CCEI-13460-2021). In addition to the ultrasound measurements, baseline measures such as history of comorbidities (hypertension, diabetes mellitus) and anthropometric measurements including weight, height and head circumference were collected averaged and analyzed.

### Ultrasound measurements

**Fig. 1:**
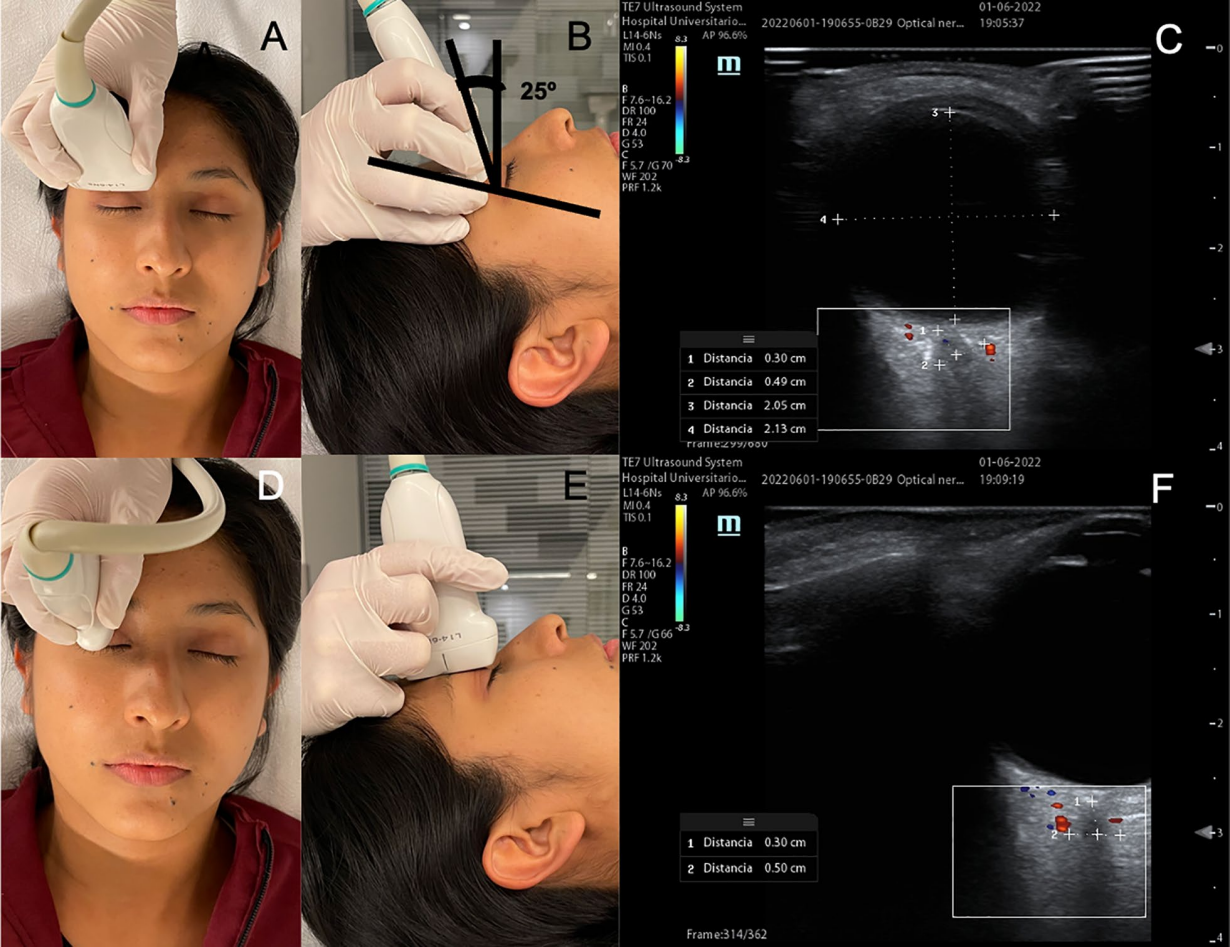
Ultrasound measurements technique. **A**–**B**: The transducer positioned on the transverse axis with an initial angle of 25° and tilted until the optic nerve and the papilla were visualized. **C**: Optic nerve and papilla with color doppler mode showing the central vessels of the retina, the ciliary vessels and measurement performed using central vessels of the retina direction and landmarks from the ciliary vessels. **D**–**E**: The transducer positioned on the vertical axis. **F**: Optic nerve and papilla with color Doppler mode showing the central vessels of the retina, the ciliary vessels and measurement performed using central vessels of the retina direction and landmarks from the ciliary vessels

Ultrasonographic measurements of the ONSD and the ONSD/ETD ratio were performed by a physician final-year critical-care resident who received specialized theoretical and practical training that certified them to perform the measurement and further evaluated by a researcher specialized in neurovascular ultrasound who performs ultrasound assessments as part of their daily practice. The measurements were obtained with a Mindray TE7 ultrasound device with a high-frequency linear transducer (7–13 MHz and the CLOSED protocol with the following parameters: frequency 10 MHz, thermal index less than or equal to 1 °C, mechanical index less than or equal to 0.23, power 20–25%, depth 40–45 mm, gain 55–60% and color Doppler frequency 1.0 kHz [12]. For patients’ safety, all measures were done less than 15 s and ultrasound images were collected as a video clip so images could be measured without long ultrasound probes, a pause of 1 min was taken between every measure, the whole protocol took around 12–15 min. The participant was placed in a supine position with the head between 15 and 25°, the protocol was started with evaluation of the right eye. The transducer was positioned on the transverse axis with an initial angle of 25° and tilted until the optic nerve and the papilla were visualized. Then, color Doppler was activated to find the central vessels of the retina, determine their direction and guide the measurement (distance from the papilla and the ONSD). Then, the ciliary vessels were identified to determine the edges of the optic nerve sheath and perform the correct measurement by freezing the image and acquiring the ETD and the ONSD 3 mm from the papilla. The procedure was repeated three times on the transverse axis; then, the transducer was positioned on the longitudinal axis, and the previous procedure (except the measurement of the ETD) was again repeated three times (Fig. 1). The entire ultrasound assessment was then performed for the contralateral eye.

### Statistical analysis

The sample size was calculated based on the estimation of the mean for an infinite population under the assumptions of a variation of 0.4 mm, an alpha error of 0.05 and Z score of 1,96, 222 subjects were needed. Continuous variables are represented as medians and interquartile ranges or means and standard deviations depending on their distribution, while categorical variables are presented as absolute frequencies and percentages. The continuous variables of the ONSD and ONSD/ETD ratio were subjected to a bivariate analysis and Pearson’s correlation to identify potential baseline data associated with the measurements, which were selected according to reports in the literature on the study of normal populations (age, sex, BMI, head circumference and the presence of chronic comorbidities). In addition, the measurements were compared between both eyes and evaluated with respect to those reported in other populations. All analyses were performed using the freely distributed R software, version 4.1.

## Results

A total of 230 individuals were analyzed, 123 women (53.4%) and 107 men (46.5%). A total of 17 individuals were excluded from the final analysis due to low-quality images and missing data. None of the quantitative variables in the analysis demonstrated a normal distribution. The baseline characteristics of interest of the study population are shown in Table 1. The average ONSD was obtained from the analysis and processing of three measurements in the transverse axis and three measurements in the longitudinal section of each eye. The mean ONSD of the right eye was 0.4492 cm (IQR 0.36–0.51), and that of the left eye was 0.4542 (IQR 0.36–0.51), The ONSD/ETD ratio was 0.2257 (IQR 0.1797–0.2566) for the right eye and 0.2280 (IQR 0.1859–0.2558) for the left eye (Table 2). In addition, the frequency of normal values was determined by dividing the ONSD data into 4 groups for analysis according to the data reported in other populations (< 0.33 cm, 0.34–0.45 cm, 0.46–0.55 cm, > 0.56 cm). As shown in Table 3, 13.04% of the participants had values  < 0.33 cm, 37.4% values between 0.34 and 0.45 cm, 38.6% values between 0.46 and 0.55 and 10.8% values greater than 0.55 cm.

**Table 1:** Baseline characteristics

Variables	Median (IQR), *n* (%)
Age	29 (IQR 25–36)
Sex	W: 123 (53,4%) M: 107 (46,5%)
BMI	24,1 (IQR 22–27)
Head circumference	56 (IQR 54–57)
Hypertension	11 (4,7%)
Diabetes mellitus	5 (2,17%)

*IQR* interquartile range, *BMI* body mass index, *W* women, *M* men

**Table 2:** Optic nerve sheath diameter (ONSD) and optic nerve sheath diameter/ transverse diameter of the eyeball (ONSD/ ETD)

	Right eye ONSD average (cm)	Left eye ONSD average (cm)	Right eye ONSD/ETD	Left eye ONSD/ETD
Mean	0.443	0.448	0.222	0.227
Median	0.449	0.454	0.225	0.228
IQR	0.36–0.51	0.36–0.51	0.17–0.25	0.18–0.25
Min	0.288	0.285	0.04	0.04
Max	0.7	0.69	0.49	0.56

**Table 3:** Optic nerve sheath diameter (ONSD) distribution in four groups according to normality reported in other populations

ONSD (cm)	*n* (%)
< 0.33	30 (13)
0.34–0.45	86 (37)
0.46–0.55	89 (38.6)
> 0.56	25 (10.8)

**Fig. 2:**
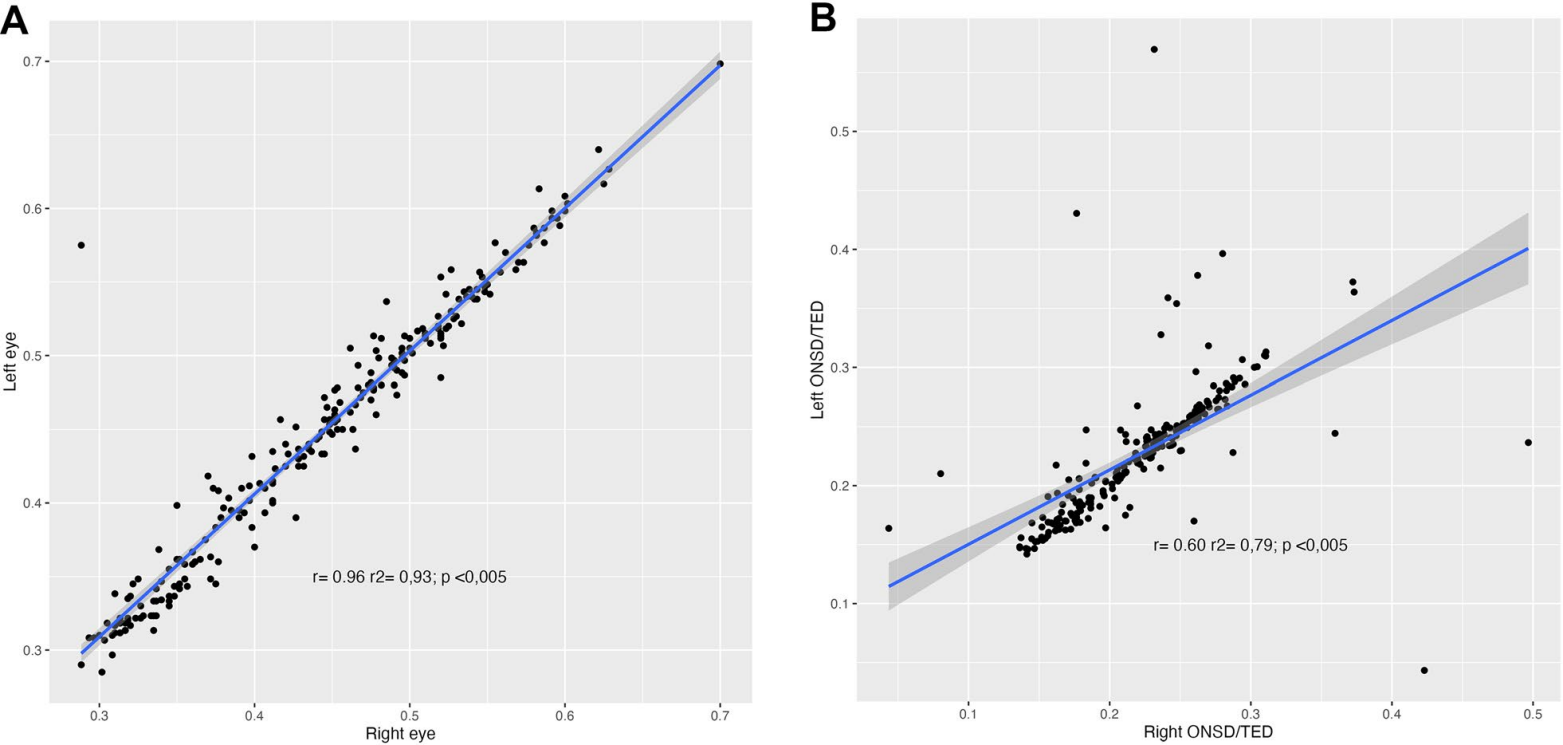
**A**: Optic nerve sheath diameter (ONSD) relation between right eye and left eye. **B**: Optic nerve sheath diameter/transverse diameter of the eyeball (ONSD/ETD) relation between right eye and left eye

**Fig. 3:**
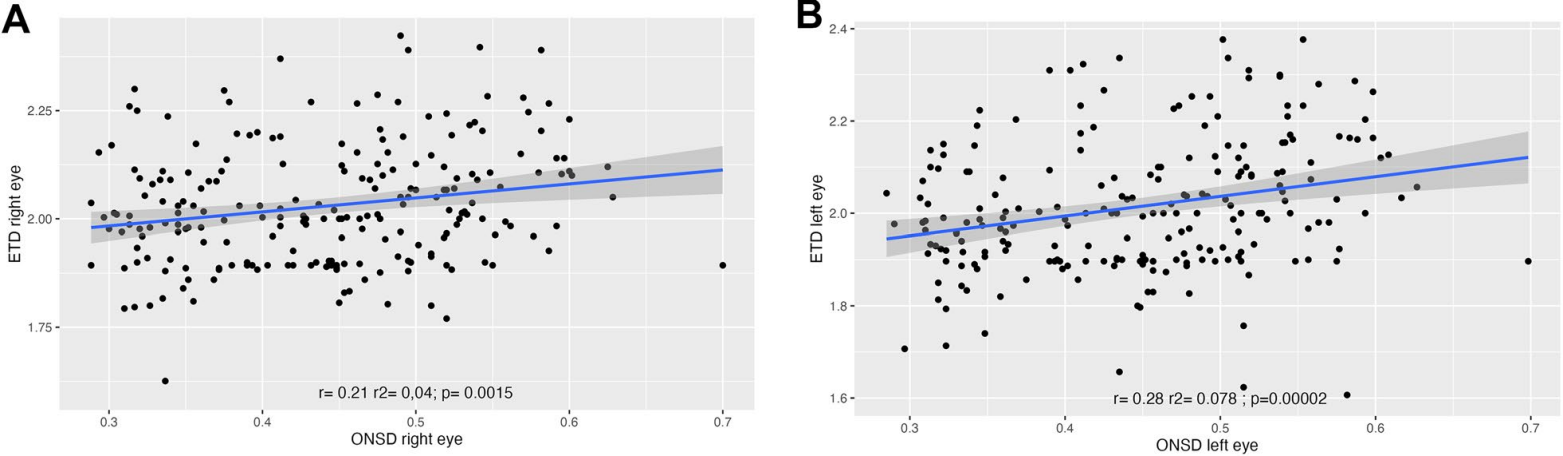
**A**: Optic nerve sheath diameter (ONSD) and ETD relation in right eye. 3**B**: Optic nerve sheath diameter (ONSD) and ETD relation in left eye

**Fig. 4:**
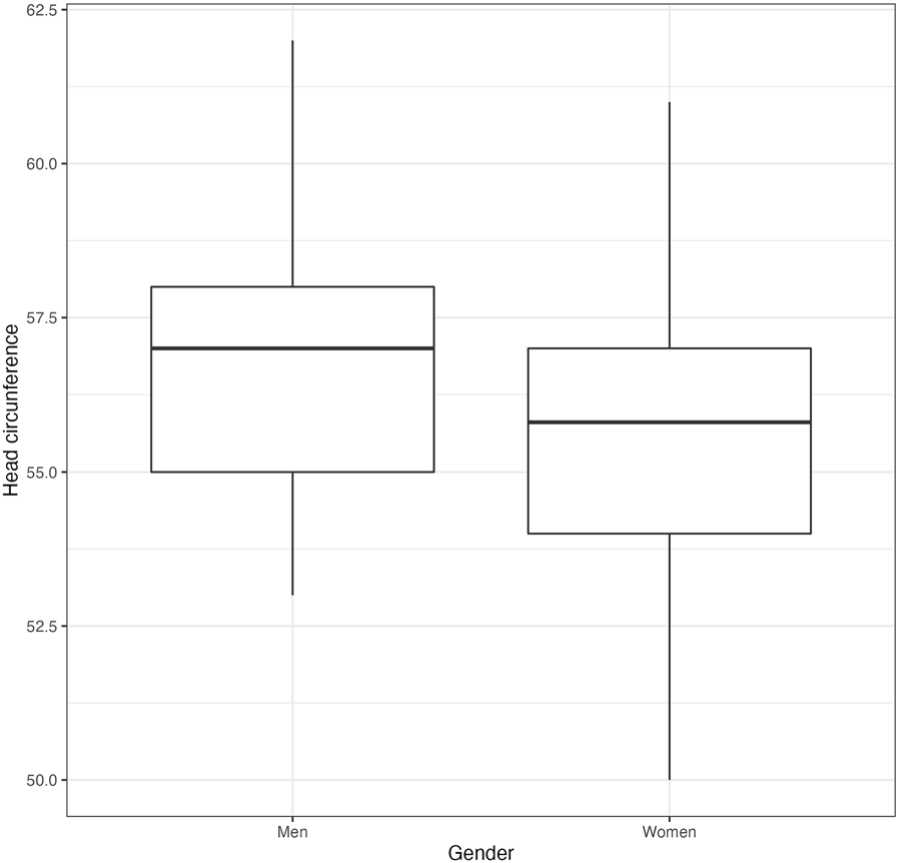
Head circumference among gender

The correlation between the measurements of the right eye and the left eye was close to 1 (ONSD, *r*^2^ = 0.93, *p* < *0.005*; ONSD/ETD ratio; *r*^2^ = 0.79, *p* < *0.005*) (Fig. 2). There was no correlation between the ONSD and the ETD (Fig. 3). History of comorbidities, sex, age, BMI, and head circumference were not correlated with ONSD AND ONSD/ETD; however, head circumference was slightly higher in men (Fig. 4).

## Discussion

The ONSD and ONSD/ETD were not correlated with baseline variables of the participating subjects, such as sex, age, BMI or even head circumference. This shows that anthropometric measurements are not confounding factors for the ONSD and ONSD/ETD at bedside.

The not normally distribution of the ONSD and ONSD/ETD with extreme values is one of the most relevant findings of the present study. 76.2% reported ONSD values between 0.34 and 0.55 cm among different populations in European, American and Asiatic countries [16], but 23,8% of the participants showed values lower than 0.33 cm and higher than 0.56 cm which has been described in healthy volunteers [16] but also in cases of hICP [18]. A total of 89% of the participants showed an ONSD less than 0.55 cm, which is the current cut-off value used in different clinical scenarios for the diagnosis of hICP [18]; however, 10.8% of the participants presented with a high ONSD value, which would nevertheless be considered normal in the context of the presumption of being healthy subjects evaluated with a standardized ultrasound measurement technique. Schroeder et.al, showed an important heterogeneity in healthy volunteers results due the ultrasound methods to measure the optic nerve sheath diameter [18]. The CLOSED protocol has been proposed as solution that guide the right machine settings in order to minimize artifacts and also apply the color Doppler for the standard evaluation of the ONSD enabling a more reliable and accurate measurement [12]. The ONSD/ETD showed similar values to reported before, but also trended to have higher values.

It is possible that the results of this study were affected by the altitude at which the city is located (2640 masl), since it has been shown that the value of the ONSD can increase by 0.14 mm per 1000 m of altitude and that adaptations can be seen within less than 1 week [17, 19, 20]. Higher ONSD values are thought to be due to increased cerebral vessel permeability at high altitudes that is not necessarily related to hICP [21]. Most of the healthy volunteers measured by many authors seems to show trends to upper values as places get higher masl level in the same country. Chen, et.al studied healthy volunteers in the Fujian province in China with an altitude of 640 masl with ONSD values up to 0.51 cm [22]. Wang, et.al also studied healthy Chinese volunteers in China but in a low-altitude province (Jilin province) with an altitude of 202 masl and found lower values of the ONSD (0.34 cm) [23]. Even so, many other studies done in European countries like Italy and Germany showed different values regardless cities altitudes (Italy: 0.48–0.62 cm) [24, 25], (Germany: 0.52–0.57 cm) [26, 27]. Also, other Asian countries like Bangladesh and Iran have measured healthy volunteers even with a wider range of values (Bangladesh: 0.42–0.47 cm) [28] (Iran: 0.31–0.33 cm) [28, 29]. The great variability of ONSD values in different studies is possibly explained by the different altitudes of the cities where they were made and the difference in measurement techniques.

The findings of the present study suggest that it would not be possible to use only the value of the ONSD or the ONSD/ETD ratio for determining the presence of hICP because, as demonstrated, high values can normally be found in the general population, even when a standardized measure is applied there is a difficulty to achieve a right measurement.

However, the ONSD and ONSD/ETD ratios of the right and left eyes were almost perfectly and well correlated, respectively, which could indicate an abnormal ICP if the values between the two eyes were asymmetric [30, 31].

The present study was limited in that the sample was not analyzed in its entirety, but guarantees adequate quality of the measurements studied with the systematic application of the CLOSED protocol and the taking of serial measurements by adequately trained personnel.

## Conclusions

The findings of the present study show that in approximately 10.8% of healthy individuals, the values of the ONSD and the ONSD/ETD ratio may be a range considered pathological. It is possible that the usefulness of these measurements in clinical practice is limited, and only repeated measurements with significant changes in the ONSD or asymmetries between the measurements of both eyes linked to clinical findings would allow the diagnosis of hICP.

The objective measures of the study were not influenced by the baseline characteristics of the patients; however, as the city is located at 2640 masl, it could be that the explanation of these high values is related to the effects of altitude, so the findings of this study should be considered mainly in cities at high altitude.

Future studies are necessary to correlate these proposed normal values for the general population in patients in the neurointensive care unit.

## Data Availability

The data used in the present study are available upon request to the corresponding author.

## Abbreviations

ONSD: Diameter of the optic nerve sheath
ETD: Eyeball transverse diameter
ONSD/ETD: Diameter of the optic nerve sheath/eyeball transverse diameter
ICP: Intracranial pressure
hICP: High intracranial pressure
US: Ultrasound
cm: Centimeters
masl: Meters above sea level

## References

[1] Monro A (1783). Observations on the structure and functions of the nervous system, illustrated with tables. Lond Med J.

[2] Raboel PH, Bartek J, Andresen M, Bellander BM, Romner B (2012). Intracranial pressure monitoring: invasive versus non-invasive methods—a review. Crit Care Res Pract.

[3] Wilson MH (2016). Monro-Kellie 2.0: the dynamic vascular and venous pathophysiological components of intracranial pressure. J Cereb Blood Flow Metab agosto de.

[4] Ordookhanian C, Nagappan M, Elias D, Kaloostian PE (2018) Management of intracranial pressure in traumatic brain injury. In: Gorbunov N, Long J (eds) Traumatic brain injury: pathobiology, advanced diagnostics and acute management, vol 177. BoD – Books on Demand

[5] Rajajee V, Vanaman M, Fletcher JJ, Jacobs TL (2011). Optic nerve ultrasound for the detection of raised intracranial pressure. Neurocrit Care diciembre de.

[6] Manouchehrifar M, Lakestani M, Kashani P, Safari S (2018). Sonographic diameter of optic nerve sheath in differentiation of ischemic and hemorrhagic strokes; a diagnostic accuracy study. Am J Emerg Med noviembre de.

[7] Gökcen E, Caltekin İ, Savrun A, Korkmaz H, Savrun ŞT, Yıldırım G (2017). Alterations in optic nerve sheath diameter according to cerebrovascular disease sub-groups. Am J Emerg Med noviembre de.

[8] Thotakura AK, Marabathina NR, Danaboyina AR, Mareddy RR (2017). Role of serial ultrasonic optic nerve sheath diameter monitoring in head injury. Neurochirurgie diciembre de.

[9] Lochner P, Czosnyka M, Naldi A, Lyros E, Pelosi P, Mathur S (2019). Optic nerve sheath diameter: present and future perspectives for neurologists and critical care physicians. Neurol Sci diciembre de.

[10] Du J, Deng Y, Li H, Qiao S, Yu M, Xu Q (2020). Ratio of optic nerve sheath diameter to eyeball transverse diameter by ultrasound can predict intracranial hypertension in traumatic brain injury patients: a prospective study. Neurocrit Care abril de.

[11] Kim DH, Jun JS, Kim R (2018). Measurement of the optic nerve sheath diameter with magnetic resonance imaging and its association with eyeball diameter in healthy adults. J Clin Neurol.

[12] Aspide R, Bertolini G, Albini Riccioli L, Mazzatenta D, Palandri G, Biasucci DG (2020). A proposal for a new protocol for sonographic assessment of the optic nerve sheath diameter: the CLOSED protocol. Neurocrit Care febrero de.

[13] Pansell J, Bell M, Rudberg P, Friman O, Cooray C. Optic nerve sheath diameter measurement by ultrasound: evaluation of a standardized protocol.:7. 10.1111/jon.12936 34555223

[14] Bender M, Lakicevic S, Pravdic N, Schreiber S, Malojcic B (2020). Optic nerve sheath diameter sonography during the acute stage of intracerebral hemorrhage: a potential role in monitoring neurocritical patients. Ultrasound J diciembre de.

[15] Lochner P, Fassbender K, Knodel S, Andrejewski A, Lesmeister M, Wagenpfeil G (2019). B-mode transorbital ultrasonography for the diagnosis of idiopathic intracranial hypertension: a systematic review and meta-analysis. Ultraschall Med—Eur J Ultrasound abril de.

[16] Kim DH, Jun JS, Kim R (2017). Ultrasonographic measurement of the optic nerve sheath diameter and its association with eyeball transverse diameter in 585 healthy volunteers. Sci Rep.

[17] Tsai TY, Gozari G, Su YC, Lee YK, Tu YK (2022). Optic nerve sheath diameter changes at high altitude and in acute mountain sickness: meta-regression analyses. Br J Ophthalmol mayo de.

[18] Schroeder C, Katsanos AH, Richter D, Tsivgoulis G, Gold R, Krogias C (2020). Quantification of optic nerve and sheath diameter by transorbital sonography: a systematic review and metanalysis. J Neuroimaging marzo de.

[19] Keyes LE, Paterson R, Boatright D, Browne V, Leadbetter G, Hackett P. Optic nerve sheath diameter and acute mountain sickness. :7. 10.1016/j.wem.2012.11.003 23425353

[20] Sikri G, Singh K (2016). Optic nerve sheath diameter and acute mountain sickness. J Ultrasound Med febrero de.

[21] Wilson MH, Wright A, Imray CH (2014). Intracranial pressure at altitude. High Alt Med Biol.

[22] Chen H (2015). Ultrasound measurement of optic nerve diameter and optic nerve sheath diameter in healthy Chinese adults. BMC Neurol.

[23] Wang L (2016). Ultrasonographic evaluation of optic nerve sheath diameter among healthy Chinese adults. Ultrasound Med Biol.

[24] Lochner P, Coppo L, Cantello R, Nardone R, Naldi A, Leone MA (2016). Intra- and interobserver reliability of transorbital sonographic assessment of the optic nerve sheath diameter and optic nerve diameter in healthy adults. J Ultrasound marzo de.

[25] Strapazzon G, Brugger H, Dal Cappello T, Procter E, Hofer G, Lochner P (2014). Factors associated with optic nerve sheath diameter during exposure to hypobaric hypoxia. Neurology 27 de mayo de.

[26] Bäuerle J, Lochner P, Kaps M, Nedelmann M (2012). Intra- and interobsever reliability of sonographic assessment of the optic nerve sheath diameter in healthy adults. J Neuroimaging enero de.

[27] Bäuerle J, Schuchardt F, Schroeder L, Egger K, Weigel M, Harloff A (2013). Reproducibility and accuracy of optic nerve sheath diameter assessment using ultrasound compared to magnetic resonance imaging. BMC Neurol diciembre de.

[28] Maude RR, Amir Hossain M, Hassan MU, Osbourne S, Sayeed KLA, Karim MR (2013). Transorbital sonographic evaluation of normal optic nerve sheath diameter in healthy volunteers in Bangladesh. PLoS ONE 2 de diciembre de.

[29] Karami M, Shirazinejad S, Shaygannejad V, Shirazinejad Z (2015). Transocular doppler and optic nerve sheath diameter monitoring to detect intracranial hypertension. Adv Biomed Res.

[30] Betcher J, Becker TK, Stoyanoff P, Cranford J, Theyyunni N (2018). Military trainees can accurately measure optic nerve sheath diameter after a brief training session. Mil Med Res diciembre de.

[31] Naldi A, Provero P, Vercelli A, Bergui M, Mazzeo AT, Cantello R (2020). Optic nerve sheath diameter asymmetry in healthy subjects and patients with intracranial hypertension. Neurol Sci febrero de.

